# Performance Measures for Alcohol and Other Drug Services

**Published:** 2006

**Authors:** Deborah W. Garnick, Constance M. Horgan, Mady Chalk

**Affiliations:** Deborah W. Garnick, Sc.D., is a professor, and Constance M. Horgan, Sc.D., is a professor and center director, both at the Center for Behavioral Health, Schneider Institute for Health Policy, Heller School for Social Policy and Management, Brandeis University, Waltham, Massachusetts. Mady Chalk, Ph.D., is director of the Center for Performance-Based Policy at the Treatment Research Institute, Philadelphia, Pennsylvania

**Keywords:** health services research, health care delivery, AOD (alcohol and other drug) abuse, AODU (AOD use) treatment method, addiction care, substance abuse services, health care quality control, performance measures, program evaluation, standards of quality, Washington Circle measures, National Outcome Measures, National Committee for Quality Assurance (NCQA), Health Insurance Portability and Accountability Act (HIPAA), continuum of care, patient information system

## Abstract

Performance measures, which evaluate how well health care practitioners’ actions conform to practice guidelines, medical review criteria, or standards of quality, can be used to improve access to treatment and the quality of treatment for people with alcohol and other drug problems. This article examines different types of quality measures, how they fit within the continuum of care, and the types of data that can be used to arrive at these measures. The Washington Circle measures—identification, initiation of treatment, and treatment engagement—are a widely used set of performance measures.

Performance measures––the methods or instruments used to evaluate the extent to which health care practitioners’ actions conform to practice guidelines, medical review criteria, or standards of quality (AcademyHealth 2004)—can be valuable tools for improving access to treatment services and the quality of those services for people with alcohol and other drug (AOD) problems.

This article first examines three important aspects of the development and use of performance measures for AOD services: a continuum-of-care framework, the types of quality measures, and the types of data from which these measures can be derived. Choices the users (e.g., State agencies or providers) make in each of these areas are determined by the users’ goals and the settings where the performance measure will be applied (Horgan and Garnick 2005). This article also describes the set of performance measures developed by the Washington Circle and widely adopted in 2003, explaining the development, testing, and current use of these measures.

## Continuum-of-Care Framework

People who are experiencing various stages of AOD disorders have a variety of needs that require attention if they are to receive optimal care. Services can be seen as falling into the following stages along a continuum of care (McCorry et al. 2000):

- *Prevention/Education:* Encompasses activities designed to raise the general awareness of AOD disorders as a major issue affecting individuals, families, and society. It also includes activities designed to target high-risk people and groups for more focused interventions.
- *Recognition:* Focuses on identifying people with AOD disorders and includes activities such as screening, assessment, and referral to treatment.
- *Treatment:* Includes a broad range of services associated with treatment and rehabilitation, such as counseling, social services, medications, testing, and coordination with other treatment resources.
- *Maintenance:* Concentrates on sustaining positive outcomes over the longer term; includes self-management and peer support strategies intended to sustain post-treatment abstinence or reductions in use, improvements in role functioning and well-being, and lifestyle changes.

Performance measures should be developed and used in each of these stages of care, because excellence in one domain at the expense of the others will result in less than optimal patient care. For example, if a health plan provides comprehensive AOD treatment services but does not screen people enrolled in the plan to identify those with AOD abuse or dependence (i.e., recognition), it is failing to provide treatment to people who need it.

## Types of Quality Measures

Health care quality is defined by the Institute of Medicine (2001) as “the degree to which health services for individuals and populations increase the likelihood of desired health outcomes and are consistent with current professional knowledge” (p. 44). Building on the classic framework proposed by Donabedian (1980), researchers have developed five categories of quality measures: structure, process, outcomes, access, and patient experience.

### Structural Measures

Structural quality measures refer to the features of a health care organization that determine its capacity to provide care, such as the existence of an electronic records system or the ratio of AOD treatment providers to clients.

### Process Measures

Process measures are used to assess how well a health care service provided to a patient, or on a patient’s behalf, adheres to recommendations for clinical practice. These recommendations are based on research evidence or consensus (i.e., the views of experts when the research evidence is lacking or inconclusive) regarding the probability that providing particular services will achieve the desired outcomes. Generally, process measures are expressed as rates, with the denominator defining a population that is of interest because of its demographic and clinical condition, and the numerator defining the subgroup receiving specific services.

Process measures are particularly important in the development of performance measures because they can be used to identify specific areas of care that may need improvement. For example, if clients are not returning after an initial service, outreach efforts could be mounted in an effort to retain clients in treatment. Moreover, the data to calculate process measures often can be obtained as part of an administrative data system that includes information on clients’ dates and types of treatment services. Recently, researchers have developed processes by which a range of stakeholders (e.g., clinicians, specialists in measurement) can work together to select core sets of process measures for common use based on analyses of how meaningful and feasible different process measures are (Hermann et al. 2004).

### Outcome Measures

These measures generally are used to evaluate the state of a patient’s health resulting from the health care services and interventions received. In general, outcomes can be considered both in terms of patient functioning and in terms of categories of symptom severity related to the patient’s clinical problem. For AOD disorders, health outcomes can be expanded to include four areas: sustained reductions in AOD use, improvements in personal health, sustained improvements in functioning (e.g., employment), and sustained reductions in threats to public health and safety (McLellan et al. 2005).

Attributing improved patient outcomes to providers’ specific actions can be difficult because outcome measures of quality reflect the cumulative impact of multiple factors such as the timeliness of services and the appropriateness of the type or amount of services for a person’s substance abuse or dependence problem. Outcomes also are influenced by factors that depend on the patients themselves and are outside the health care system, such as the choices the patients make (e.g., to remain in treatment for the full course of recommended services); their living situation, income, and employment; or whether the patients have other medical conditions.

### Access Measures

Access measures assess the extent to which a person who needs care and services is able to receive them. This type of measure is particularly important for addictive disorders because of the large gap between those who have serious problems and those who actually use any services.

### Patient Experience

These measures are aggregated from patients’ reports about their observations of and participation in AOD treatment.

## Data Sources for Performance Measures

Sources of data for performance measures include medical records, patient surveys, and administrative data used to pay bills or to manage care in health plans, government programs, and State substance abuse systems.

### Medical Records

Medical records provide detailed information on clinicians’ observations, impressions, thought processes, and referrals to other providers; and on patients’ tests, results, medications, and reactions to clinicians’ advice. A difficulty with using medical records as a data source, however, is that patients often receive services in multiple sites in the specialty substance abuse, mental health, and general medical sectors, and each of these sites generates its own set of medical records. Unfortunately, it is difficult for various segments of the health care system or for behavioral health treatment facilities to share electronic medical records (McLellan et al. 2003), primarily because the various providers do not share a vocabulary or other established industry standards that would guide the development of effective computerized medical record data systems. Thus, medical records are not commonly used for performance measures because of the cost and complexity of compiling the necessary data from a wide range of medical record formats.

### Patient/Enrollee Surveys

Surveys can capture information on how patients view their treatment experience, including their satisfaction with care and its various treatment components or events (e.g., being offered information about a medication’s side effects) and their perceptions about the outcomes of care. Performance measures based on patient experience specific to addiction treatment still are being developed, but two major efforts are worthy of mention.

*The Experience of Care and Health Outcomes (ECHO) Survey* (CAHPS Survey Users Network 2004) includes questions on a wide range of issues, such as access to care, experience of providers’ communication and listening skills, satisfaction with care, and perceived benefits of treatment. From 2002 through 2004, the National Committee for Quality Assurance (NCQA) adopted a version of this survey for commercial managed behavioral health care organizations, though not for general health plans (CAHPS Survey Users Network 2004).

*The Modular Survey Initiative*, sponsored by the Substance Abuse and Mental Health Services Administration (SAMHSA), is designed to develop and promote a set of consumer perception-of-care survey tools that can be widely used across settings and populations (Doucette 2004; Merrick 2004; Smith 2004). The modular survey consists of a core set of items addressing quality of care, access to services, and perceived improvement as a result of having received services. Additional modules addressing the quality of the helping alliance, motivation to change, and relapse/recovery status are under development. The modular framework increases the flexibility of the survey in that some survey modules are appropriate for the full population range and others are specific to a particular type of service (e.g., AOD disorders or mental health), age group (e.g., youth or adults), service settings (mental health/AOD), and delivery/financing vehicles (i.e., public or private, insured or uninsured). Table 1 shows examples of the survey’s domains and survey items within those domains.

### Administrative Data

In the course of providing and paying for care, insurers and related organizations generate administrative data on the characteristics of the population they serve as well as the utilization of and charges for services, often at an individual-user level. Even without full standardization of data collection, these data usually contain certain key elements, such as the following (Garnick et al. 2002*a*):

- Amount billed and amount reimbursed, often separately by service
- Type of service
- Number of units (e.g., days of service)
- Location of service
- Diagnosis and procedure codes for clinical services.

Patients receiving AOD-related services can be identified and their care tracked over time and across settings via administrative data. Large amounts of data are available, information already is collected by organizations that are familiar with its use, and data usually are collected with common data elements and formats.

Insurance claims data, one type of administrative data, have been used for measuring the quality of AOD services in a variety of settings, including commercial health plans and the Federal Government’s Medicare and Medicaid programs. In addition, systems such as staff-model managed care plans, which directly manage and pay physicians, as well as State-run specialty hospitals and Department of Veterans’ Affairs (VA) facilities, deliver care through facilities they own and providers they employ. Most of these systems generate encounter-level records that have dates and descriptions of services, such as are included in claims.

Data generated by publicly funded mental health and AOD-treatment systems administered through State departments of public health, mental health, or substance abuse could be useful for performance measurement. Across the 50 States, the availability of data to support performance measurement varies widely, however. Some States are leaders in data systems and have detailed data on date of service, types of services, and diagnoses for specific clients, but many still report only aggregate service-use statistics or the start and end dates of episodes of care without specific detail on dates for all services. More States are moving toward designing systems to accumulate the level of detail required for many performance measures.

Despite the many advantages of administrative data, this data source also has quality problems. AOD diagnoses may not be coded accurately or completely because of issues of insurability, confidentiality, stigma, or even lack of space on the claims forms to record multiple diagnoses. Furthermore, codes for procedures specific to the treatment of addictions are not included in the Current Procedural Terminology (CPT), which is the most widely used coding system for procedures. (The CPT is published by the American Medical Association and updated annually.) A common coding practice, for example, is to indicate a substance use disorder diagnosis along with a generic psychiatric procedure code that does not differentiate between services for mental health problems and substance use disorders. This makes calculating performance measures much more complex.

The Health Insurance Portability and Accountability Act (HIPAA) contains electronic transaction regulations requiring a national procedure code set. These requirements, which were implemented as of 2003, could solve the problem posed by the lack of treatment codes for services related to substance use disorders. Not only has HIPAA standardized codes for procedures and durations of treatment, but the services code set has been expanded to more precisely reflect the wide range of existing types of services for substance use disorders (Centers for Medicare and Medicaid Services 2005; SAMHSA 2004). This code set, Level II of the Healthcare Common Procedural Coding System (HCPCS), is used to identify items and services, including behavioral services that are not included in the CPT codes. See the textbox for a list of selected items from this expanded code set.

## Developing Performance Measures: The Washington Circle Example

In 1998, the Center for Substance Abuse Treatment convened the Washington Circle, a multidisciplinary group of providers, researchers, managed care representatives, and public policymakers, to address the need for performance measures for people with AOD disorders (McCorry et al. 2000). Building on the foundation laid by several other groups in both the public and private sectors, this group outlined a set of seven performance measures that can apply to all four stages of the continuum of care––prevention/education, recognition, treatment, and maintenance.

During 1999, the Washington Circle selected three of these measures, defined in detail below, for initial focus. From 1999 through 2002, the group wrote technical specifications and carried out pilot testing (Garnick et al. 2002*b*). The measures were applied first in commercial managed care plans that had enrolled populations and administrative datasets which used common definitions and contained detailed data on services and dates of enrollment. The Washington Circle further collaborated with the National Committee for Quality Assurance to refine the measures. In 2004, NCQA adopted the measures for inclusion in its Health Plan Employer Data and Information Set (HEDIS^®^) (NCQA 2006). HEDIS is an information system that tracks quality of care in health plans, although the core HEDIS measures also are used more widely, beyond their formal scope.

**The Healthcare Common Procedural Coding System, Level II**

Level II of the Healthcare Common Procedural Coding System (HCPCS) is a standardized coding system that is used primarily to identify products, supplies, and services not included in the CPT codes, including behavioral services. A full listing of the HCPCS Level I codes (Current Procedural Terminology [CPT] codes) and Level II codes can be found on the SAMHSA Web site. The first seven of the approximately 100 HCPCS Level II codes are shown below:

**Table:** 

H0001	Alcohol and/or drug assessment
H0002	Behavioral health screening to determine eligibility for admission to treatment program
H0003	Alcohol and/or drug screening; laboratory analysis of specimens for presence of alcohol and/or drugs
H0004	Behavioral health counseling and therapy, per 15 min.
H0005	Alcohol and/or drug services; group counseling by a clinician
H0006	Alcohol and/or drug services; case management
H0007	Alcohol and/or drug services; crisis intervention (outpatient)

SOURCE: Substance Abuse and Mental Health Services Administration (SAMHSA) 2005*a*.

### Definitions of Three Tested Performance Measures

The three performance measures chosen for testing by the Washington Circle were identification, initiation of treatment, and treatment engagement. (For detailed specifications of which diagnoses are included, how to calculate new episodes, or other details necessary to consistently calculate the performance measures across sites, see the Washington Circle Web site [www.washingtoncircle.org] or NCQA 2006.)

#### Identification of AOD Services

This measure captures the percentage of a health plan’s enrollees that receive any addiction-related services; it falls into the recognition stage of the continuum of care (described above). If health plans actively screen their enrollees for AOD problems, and if providers code substance abuse or dependence diagnoses more accurately, the health plan likely will score higher on this measure. Specifically, this measure reports the percentage of health plan members with at least one claim containing a diagnosis of AOD abuse or dependence and services related to the treatment of these diagnoses during the measurement year.

#### Initiation of Treatment

The initiation of treatment measure captures information on patients with substance abuse or dependence diagnoses who return for additional services. This is a process measure that falls into the treatment domain on the continuum of care. Specifically, it is defined as the percentage of adults diagnosed with a new episode of AOD abuse or dependence and receiving a related service who either (1) initiate treatment through an inpatient AOD admission or (2) have an initial outpatient service for AOD abuse or dependence and receive any additional AOD services within 14 days.

#### Treatment Engagement

This is an intermediate step between initially accessing care (in the first visit) and completing a full course of treatment. This measure is defined as the percentage of adults diagnosed with AOD abuse or dependence who receive two additional AOD treatments within 30 days after initiating treatment.

### Key Decisions in Developing the Washington Circle Measures

Defining a service that begins a new episode of care is essential for many performance measures (Hornbrook et al. 1985). Both the initiation and engagement measures rely on finding people with new episodes of AOD services; this is the population of interest, and they are represented by the denominator. To qualify as a new episode, there must be a period of 60 days, referred to as a “negative diagnosis history,” during which the person had no claims or encounters associated with any diagnosis of AOD abuse or dependence. This period without any claims also is called a “clean” period in the literature on episodes of care. An analysis of adults who were commercially insured showed that extending this negative diagnosis period from 60 to 90 days reduced the number of new AOD episodes, as expected, but the initiation and engagement rates did not change markedly (Horgan et al. 2004). Thus, this analytic decision reflects a trade-off between having a larger number of people included in the measure and having more confidence that the people selected actually are beginning new episodes of treatment.

In developing these measures, the Washington Circle gave special consideration to some services. In particular, the group decided that detoxification and emergency room care could mark the beginning of a treatment episode but were not appropriate for continuing care, as these services do not by themselves constitute treatment for AOD disorders. In addition, participation in Alcoholics Anonymous and other community support fellowships was not included in the measures, in part because data are not collected on participation. Moreover, although these groups clearly are important for the continuation of treatment-initiated sobriety and social function, by their own charters they are neither structured to provide the professional services that so often are needed to stabilize psychologically and emotionally unstable patients, nor designed to address the complications of addiction which can interfere with behavioral change (McCorry et al. 2000).

### Applications of Washington Circle Performance Measures

Several groups have calculated the three performance measures discussed here––identification, initiation, and engagement––throughout the testing and development process and in real-world applications. Although these measures were developed for commercial health plans, they also have been used with a State data system, VA data, and Medicaid data. The applications described here share common themes. They all demonstrate the use of data that currently are collected for administrative purposes to monitor quality, and they all build on the performance measures for AOD services developed by the Washington Circle and adopted by NCQA in 2004.

#### Commercial Health Plans

Table 2A compares AOD identification, initiation of treatment, and treatment engagement rates for commercial health plans using three data sources:

- The Washington Circle pilot test using data from six managed care plans (the identities of which are confidential).
- 2001 data from Marketscan^®^, a large database maintained by Medstat, which consists of claims submitted from private health insurance companies and managed care vendors across the United States for about a quarter million adults age 18 and older.
- Data compiled by NCQA for the first year that the measures were used by health plans nationwide.

These measures also were used to compare the periods before and after the Federal Employees Health Benefits Program implemented parity for six health plans (data not shown) (Northrop Grumman Information Technology 2004). Although the performance rates vary considerably, some common themes emerge. The identification rates are all under 1.5 percent, which is considerably less than the approximately 5 percent of commercially insured adults estimated to need treatment for substance abuse or dependence. Initiation rates ranged from 26 to 46 percent, and engagement rates ranged from 14 to 49 percent.

#### The Public Sector

Table 2B compares the same performance measures shown in Table 2A using three data sources from the public sector: VA data (Harris et al. 2005), data from the Oklahoma Department of Mental Health and Substance Abuse Services (2005), and Medicaid data from more than 40 States (Tompkins and Reif 2005).

Because researchers are still collaborating with States to adapt the Washington Circle measures to populations served by public funds, the results shown in the table are intended to be illustrative. The public sector identification rates are higher than in the commercial health plans, ranging from 6.3 percent in the VA to 9 percent in the Oklahoma State system. Comparing rates for various populations requires great caution, however, because types of data and data collection methods vary. For example, as a State mental health and substance abuse system, Oklahoma does not have an enrolled population, so administrators elected to use adults with family incomes less than twice the Federal poverty level (i.e., under 200 percent of poverty) as an estimate of the population their treatment system serves, and thus as the denominator for the identification rate. For initiation and engagement rates, the Medicaid analysis includes services provided in both specialty and in general medical settings, whereas the Oklahoma results are limited to specialty settings.

Two important considerations in relation to using performance measures in both the public and private sectors are how to interpret the data and how to avoid setting up incentives that encourage a health plan to focus on one measure at the expense of others. In terms of interpretation, data on performance measures should be interpreted with care. For example, if a health plan has a high identification rate, it may be appropriately identifying people, but some of these people may be more difficult to motivate into treatment. Thus, the plan would show a lower engagement rate. A low engagement rate could be explained by the health plan as resulting from barriers to continued care, such as limited referral networks or complex approval systems. In terms of incentives, if a health plan offers incentives for improving one particular performance measure (e.g., identification), that measure may improve through increased screening, provider education, or outreach to enrollees, for example, but at the expense of the other two measures (e.g., treatment initiation and engagement).

## Future Directions

Performance measures are potentially powerful tools for commercial health plans, State providers (through Medicaid programs and State-run treatment systems), and the VA to use in their efforts to improve the quality of care for people with substance use disorders. Currently, commercial health plans are being held accountable for improving their baseline AOD services because performance measures have been included in the HEDIS national reporting system described earlier. States generally fund AOD treatment through Medicaid programs and through Substance Abuse Prevention and Treatment (SAPT) block grants. These block grants focus on State accountability by requiring States to measure current performance, set targets, and make changes to meet these targets for delivery of AOD services.

Certainly, many health plans and public payers have developed performance measures focused on services for substance use disorders as part of their internal quality improvement initiatives. For example, the VA annually publishes the results of seven performance indicators showing data both nationally and for specific facilities and networks (Harris et al. 2005). Oklahoma has developed a regional performance management report to offer quarterly feedback to providers, consumers, department administrators, and other stakeholders on key indicators of the performance of AOD and mental health treatment providers (Oklahoma Department of Mental Health and Substance Abuse Services 2005).

This article has focused on performance measures that are being developed at a national level for widespread use. The three Washington Circle measures described, although developed to be applied at the health-plan level, also may be useful for quality improvement efforts at the local or facility level. For example, to pinpoint areas for improvement, health plans might break down information on initiation and engagement by age, gender, provider group, location, type of substance used, employment status (i.e., employee or dependent), or initial care by specialty or general medical providers. States might provide prompt feedback on initiation and engagement rates to allow facilities to track improvement efforts.

The development of performance measures for AOD services still is at an early stage, and ongoing efforts are needed to create measures that encompass all four domains––prevention/education, recognition, treatment, and maintenance––originally envisioned by the Washington Circle. In addition, ongoing resources are required to do the following:

- Conduct research to link process measures explicitly to outcomes.
- Update measures as new evidence on treatment effectiveness emerges and new ways of conceptualizing addiction are developed.
- Monitor the opportunities afforded by ongoing efforts for the improvement of administrative data systems, linkage across multiple sources of services, and new electronic medical records.
- Disseminate measures that are meaningful to frontline providers (Valenstein et al. 2004).

As new data sources such as electronic medical records are used more commonly, it may become feasible to combine performance measure concepts with evidence-based practice guidelines that are more focused on specific treatment and allow for provider discretion (Garber 2005). For example, practice guidelines may include information on selecting an appropriate referral for a patient’s clinical condition, whereas performance measures indicate whether the patient received followup services but do not give details about whether it was the right service.

SAMHSA’s new set of National Outcome Measures focused on AOD treatment and prevention is designed to facilitate achievement of the overarching goal of providing reliable and valid assessment of important dimensions of service system performance that can be useful at the national, State, and local levels (SAMHSA 2005*b*).

Performance measures are a starting point in quality improvement for people who do not receive adequate treatment for their addiction problems. These measures can be powerful tools for drawing attention to deficits in the current treatment system, monitoring the effectiveness of efforts to improve quality, designing incentives for quality improvement, and targeting areas where quality improvement is needed.

## Figures and Tables

**Table 1:** Examples of Domains and Survey Items Developed for the Modular Survey Initiative

Domain	Item	Disagree	Somewhat Agree	Agree	Strongly Agree	Does Not Apply
Quality/Access	When I needed services right away, I was able to see someone as soon as I wanted.	□	□	□	□	□
Quality/Access	The people I went to for services spent enough time with me.	□	□	□	□	□
Helping Alliance	I talk freely, openly, and honestly with my counselor.	□	□	□	□	□
*As a result of the treatment or services I received . . *.						
Functional Status	I am better able to cope when things go wrong.	□	□	□	□	□
Functional Status	I am not likely to use alcohol and other drugs.	□	□	□	□	□
Problem Recognition	I see that using alcohol and/or other drugs is a problem for me.	□	□	□	□	□
Relapse Recovery	I know how to stay away from situations that lead me to drink or use drugs.	□	□	□	□	□

SOURCE: Doucette 2004.

**Table 2:** Comparison of Rates of Adult AOD Identification, Initiation of Treatment, and Treatment Engagement for Commercial Health Plans and Public Sector Data Sources

	2A. Commercial Health Plans			2B. Public Sector		
		
	Washington Circle Pilot Testing	Marketscan^®^	National Committee for Quality Assurance	Veterans Affairs	Oklahoma Department of Mental Health and Substance Abuse Services	Medicaid
	
Year	1997–2000	2001	2003	FY 2004	FY 2005	1999
Identification	0.7%–1.4%	0.46%	0.8%	6.3%	9%	2.7%
Initiation	26%–46%	47%	45%	26.2%	75.3% after outpatient 16.6% after detox 18% after residential	25%
Engagement	14%–49%	12%	16%	8.8%	61% after outpatient 9% after residential	14%

SOURCES:

Washington Circle Pilot Testing: Garnick et al. 2002*b*.

Marketscan^®^: Analysis of authors, with Kay Miller and Margaret Lee.

National Committee for Quality Assurance rates: Horgan et al. 2004.

Veterans Affairs: Harris et al. 2005.

Oklahoma Department of Mental Health and Substance Abuse Services: Oklahoma Department of Mental Health and Substance Abuse Services 2005.

Medicaid: Analysis of 1999 Medicaid data for all States except Hawaii from the Medicaid Statistical Information System by Christopher Tompkins and Sharon Reif, Center for Behavioral Health, Heller School for Social Policy and Management, Brandeis University.
